# Petersen’s space hernia

**DOI:** 10.11604/pamj.2021.38.5.25589

**Published:** 2021-01-05

**Authors:** Danilo Coco, Silvana Leanza

**Affiliations:** 1 Department of General Surgery, Ospedali Riuniti Marche Nord, Pesaro, Italy,; 2 Department of General Surgery, Carlo Urbani Hospital, Jesi (Ancona), Italy

**Keywords:** Petersen’s space hernia, morbid obesity, Italy

## Image in medicine

Petersen’s space hernia was first described in 1900 by Walther Petersen MD. It is an internal hernia, arising after any type of gastrojejunostomy, in particular, after Roux-en-Y anastomosis. A 49-years-old Caucasian woman was admitted to Emergency Room of our institution complaining of epigastric abdominal crampy pain and alimentary vomiting. Her past medical history pointed out Roux-Y Gastric Bypass for morbid obesity and weight loss of 60kg. Her abdominal physical examination revealed Blumberg sign. Vital signs were normal. White blood tests were in normal range. Body temperature was 36.2°C. CT scan demonstrated a “twist” in the mesentery with “whirlpool sign” of the jejunum. The patient was referred to operating theatre. We performed an exploratory laparotomy, disclosing an incarcerated Petersen’s space hernia of the common limb, with obstruction of the jejunum. The incarcerated bowel was repositioned and there was no irreversible ischemia, no resection being required. The Petersen's space was closed with a suture. The post-operative period was unremarkable and the patient was discharged at the fourth post-operative day.

**Figure 1:**
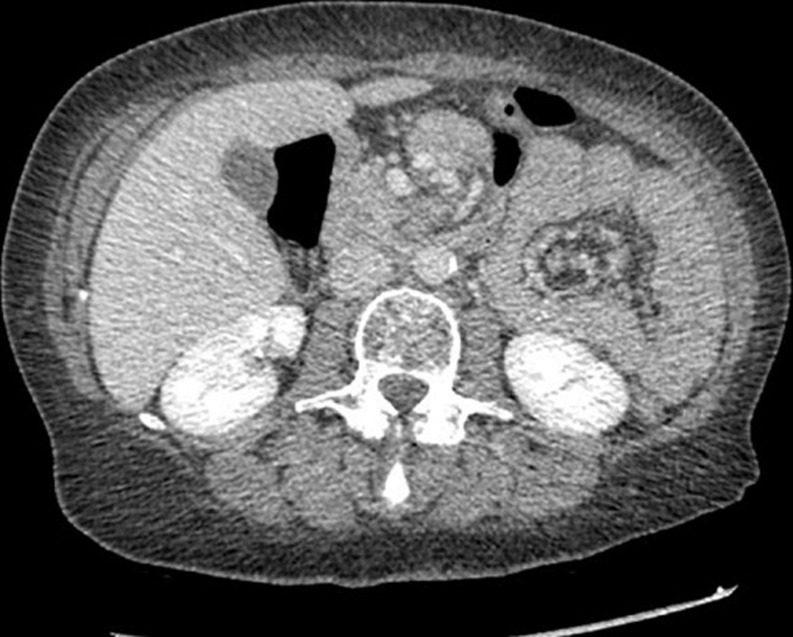
CT scan demonstrated a “twist” in the mesentery with “whirpool sign” of the jejunum

